# Anodal Electrical Taste Stimulation to the Chin Enhances the Salt Taste Perception in Subarachnoid Hemorrhage Patients

**DOI:** 10.7759/cureus.56630

**Published:** 2024-03-21

**Authors:** Masahito Katsuki, Taiki Fukushima, Tetsuya Goto, Yoshiki Hanaoka, Naomichi Wada, Takuya Nakamura, Shiori Sasaki, Tetsuyoshi Horiuchi

**Affiliations:** 1 Department of Neurosurgery, Japanese Red Cross Suwa Hospital, Suwa, JPN; 2 Neurology, UBeing, Inc., Nagoya, JPN; 3 Department of Neurosurgery, Shinshu University School of Medicine, Matsumoto, JPN

**Keywords:** telemedicine, virtual reality, subarachnoid hemorrhage, stroke, salt reduction, hypertension, elderly, anodal electrical taste stimulation

## Abstract

Aneurysmal subarachnoid hemorrhage (SAH) is a critical condition associated with high mortality rates. Hypertension is a significant risk factor for SAH development and recurrence following coil embolization for a ruptured aneurysm. While reduction of salt consumption is crucial for managing hypertension, it often compromises food taste. Anodal electrical taste stimulation (ETS) has been proposed to enhance taste perception without altering salt content. We present the case of a 69-year-old female SAH patient with a ruptured aneurysm at the anterior communicating artery who underwent coil embolization and in whom we tested ETS’s efficacy in enhancing the salt taste perception on day 42 after the procedure. ETS effectively enhanced the salt taste perception threshold and perceived concentration; the threshold for salt taste without electrical stimulation was 0.8% of salt-impregnated filter paper, whereas that with electrical stimulation was 0.6%. The perception of salt taste was enhanced: 0.8% and 1.0% of filter papers were perceived as 0.6% and 0.8% without electrical stimulation and 1.0% and 1.2% with electrical stimulation, respectively. This is the first report describing the salt perception-enhancing effect of ETS in an actual patient. Further studies involving actual patients are required to determine how ETS affects habitual salt intake and blood pressure trends.

## Introduction

Aneurysmal subarachnoid hemorrhage (SAH) can be a fatal condition, and only about 30% of patients have a favorable prognosis [1]. Transcatheter coil embolization for ruptured aneurysm is a less invasive procedure, but coil embolization has been generally associated with a higher incidence of post-treatment rupture and aneurysm recurrence compared to clipping [2]. Hypertension is recognized as a major risk factor for the development, enlargement, and rupture of cerebral aneurysm [3], as well as recurrence [4] and death [5] after coiling. Therefore, SAH patients need to undergo sufficient antihypertensive therapy over the long term after coil embolization to avoid aneurysm recurrence or death.

Reducing salt intake is an essential non-drug treatment for hypertension. Reducing 1 gram of salt per day lowers blood pressure by 1 mmHg [6]. Japanese guidelines for managing hypertension recommend that the daily salt intake should be less than 6 grams [7]. However, healthier salt-reduced foods generally are often inferior in taste due to the low concentration of taste components, necessitating the development of methods to reduce salt intake without compromising taste.

Anodal electrical taste stimulation (ETS) can potentially resolve this health-taste trade-off. ETS involves a non-invasive electrical stimulation of sensory nerves that induces, inhibits, or enhances taste sensation [8]. Thus, ETS could help individuals have healthy and tasty foods by modulating the sense of taste without physical changes in the concentration of taste-related materials. ETS’s efficacy in enhancing salt taste perception has been demonstrated among healthy volunteers [9], but it has never been studied in SAH patients. We discuss the case of an SAH patient in whom we tested the efficacy of the ETS apparatus for enhancing salt taste perception.

## Case presentation

**Figure 1:**
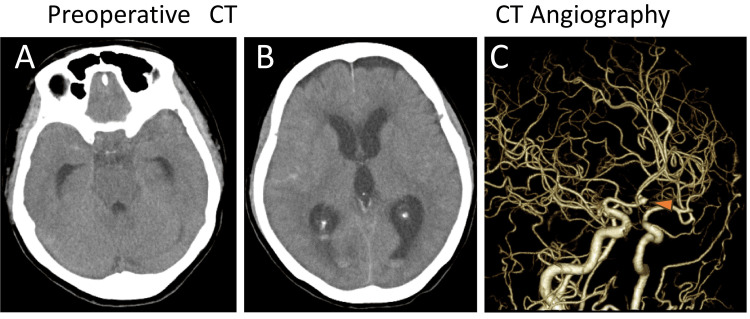
Preoperative images A CT scan confirmed SAH (A and B). CT angiography revealed a 5-millimeter ruptured aneurysm at the anterior communicating artery (orange arrowhead in C) CT: computed tomography

A 69-year-old female presented to the emergency department with sudden-onset severe headache, nausea, and vomiting. She had been treated for dyslipidemia with atorvastatin but never had been diagnosed with hypertension. She reported that her daily blood pressure was around 125/70 mmHg before the SAH onset. She never smoked and did not habitually drink. Her blood pressure at the emergency department was 168/81 mmHg. Her Glasgow Coma Scale score was E4V5M6 (total 15), corresponding to World Federation of Neurosurgical Societies Grade I, and no paresis was found in the extremities. A CT scan confirmed SAH with Fisher group 3 (Figures 1A, 1B).

**Figure 2:**
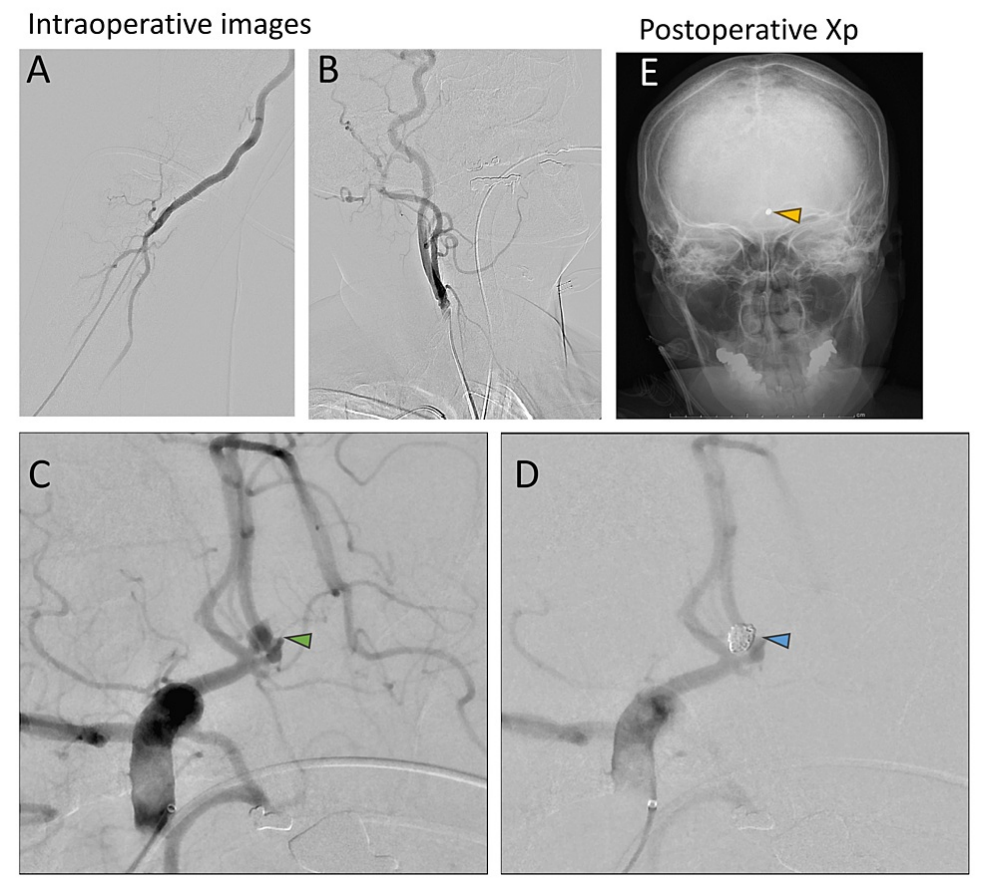
Intraoperative images The patient underwent emergent coil embolization with general anesthesia by radial artery approach (A and B). The aneurysm (green arrowhead in C) was not seen after coil embolization (blue arrowhead in D). Postoperative X-ray confirmed the presence of coils (yellow arrowhead in E)

CT angiography revealed a 5-millimeter ruptured aneurysm at the anterior communicating artery (Figure 1C). After we started antihypertensive therapy with nicardipine and sedation with propofol, the patient underwent emergent coil embolization under general anesthesia. The coil embolization involved using six coils by the radial artery approach (Figure 2A-2E) [10,11].

**Figure 3:**
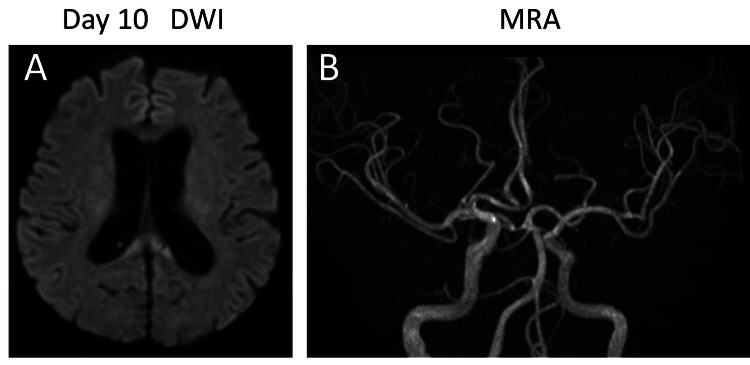
Postoperative images Angiographical vasospasm and symptomatic delayed cerebral ischemia were not observed in DWI (A) and (MRA) DWI: diffusion-weighted imaging; MRA: magnetic resonance angiography

On postoperative day one, the patient was found to have hydrocephalus, and we started continuous spinal drainage until day 13. Cerebral vasospasm prevention was performed with clazosentan and fasudil infusion therapy. Angiographical vasospasm and symptomatic delayed cerebral ischemia were not observed (Figures 3A, 3B).

The patient's mini-mental state examination score was 29/30, and her frontal assessment battery score was 15/15 on day 21. After undergoing rehabilitation for SAH and its related disuse syndrome, she was discharged on day 45 with a modified Rankin Scale score of 0 (no neurological deficits). Amlodipine 5 milligrams was started during this hospitalization to treat hypertension, and her blood pressure has been maintained around 110/70 mmHg, down from 130-140/75-85 mmHg after surgery. We will carefully monitor whether antihypertensive medications should be continued for her on an outpatient basis.

Methods of two experiments

On day 42, we conducted two psychophysical experiments to demonstrate the enhancement effect of ETS on salt taste perception. Objective taste or smell dysfunction was not considered at the time of experiments. In all experiments, we followed the safety standards used in existing reports [9]. All experiments were conducted by adhering to the safety standards approved by the local ethics research committee of The Japanese Red Cross Suwa Hospital Ethics Committee (2023-5-2). Before participation, the experiment was explained to the patient, and she signed consent letters. The study complied with the ethical standards of the Declaration of Helsinki.

**Figure 4:**
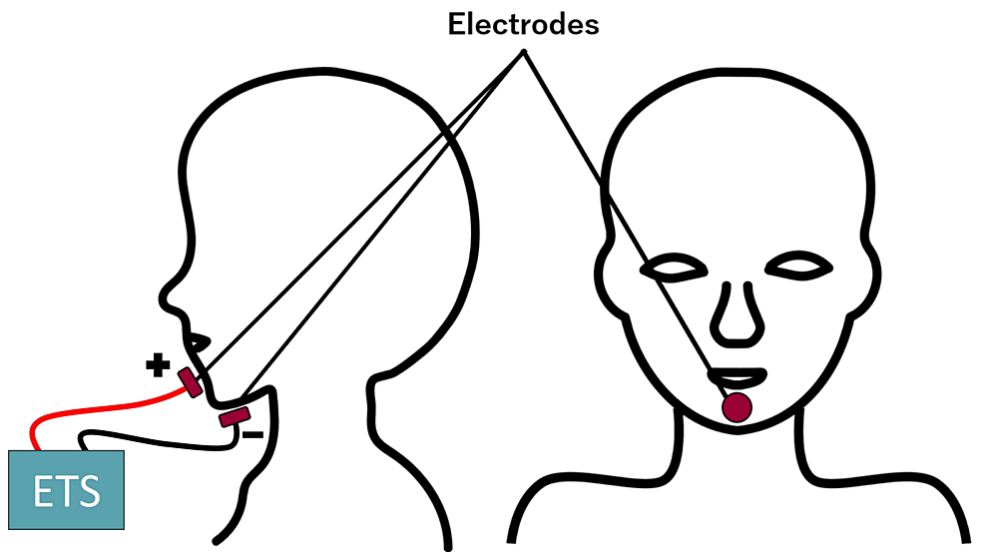
Electrodes arrangement Electrodes were attached around the center of the chin (anode) and the anterior part around the hyoid bone (cathode). A direct current of 1.0 mA was passed using ETS during the experiment ETS: electrical taste stimulation Image credits: Taiki Fukushima

Electrodes were attached around the center of the chin (anode) and the anterior part around the hyoid bone (cathode), as shown in Figure 4.

**Figure 5:**
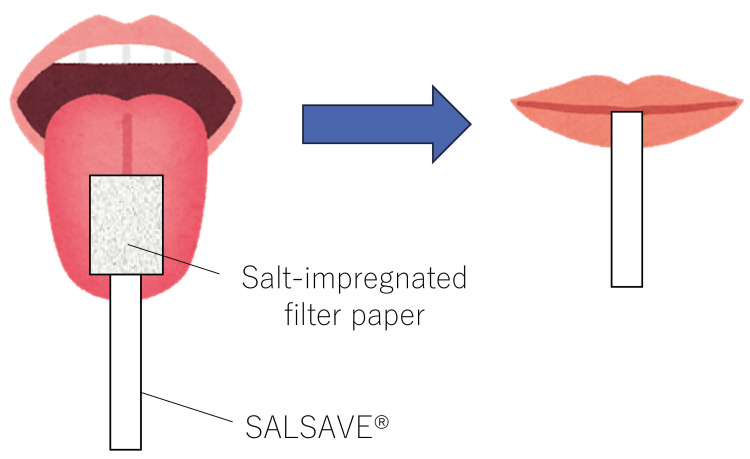
Taste examination with SALSAVE® SALSAVE^®^ is composed of a set of seven filter papers with different salt concentrations (0%, 0.6%, 0.8%, 1.0%, 1.2%, 1.4%, and 1.6%). Each filter paper was placed on the tongue, and the taste is usually evoked in about three seconds when the mouth is closed Image credits: Taiki Fukushima

In experiment 1, the patient was seated on a chair and instructed to check the salt taste strength of salt-impregnated test papers (SALSAVE®, 07830010, Advantech, Toyama, Japan). SALSAVE® is composed of a set of seven filter papers with different salt concentrations (0%, 0.6%, 0.8%, 1.0%, 1.2%, 1.4%, and 1.6%). Each filter paper was placed on the tongue, then the mouth closed (Figure 5).

The filter paper test was started with the lowest salt concentration to determine the first paper that invoked the salty taste, which was the taste threshold. After rinsing the mouth thoroughly and resting for at least 10 minutes, the same procedure was done with a direct current of 1.0 mA by ETS, and the threshold was rechecked. Experiment 1 was performed without informing the patient about the concentration. This salt taste threshold test method has been widely performed [12].

After rinsing the mouth thoroughly and resting for at least 10 minutes, we performed experiment 2. After asking the patient to remember the seven concentration levels of SALSAVE®, she was given 0.8% filter paper without revealing the concentration. She was asked about the concentration levels of the two filter papers (from 0.6% to 1.6%) in the absence of electrical stimulation. After rinsing the mouth thoroughly and resting for at least 10 minutes, the same filter papers were given to the participant while a 1.0 mA direct current was applied, and she was asked as to which concentration she found. We then gave her 1.0% filter paper, and the same procedure was repeated. This meant that this experiment was performed with and without electrical stimulation at two different concentrations. Similar experiments about such concentrations have been reported in the literature [13].

Results of experiments

In experiment 1, the taste threshold for any taste and salt taste without electrical stimulation were 0.6% and 0.8%, and those with electrical stimulation were 0.6% and 0.6%, respectively. In experiment 2, the perception of salt taste was enhanced: 0.8% filter paper was perceived as 0.6% without electrical stimulation and 1.0% with electrical stimulation; 1.0% filter paper was perceived as 0.8% without electrical stimulation and 1.2% with electrical stimulation. No adverse events related to ETS were observed.

## Discussion

This is the first case report to describe ETS-enhanced salt taste perception in an SAH patient, which contrasts with previous reports involving healthy volunteers. We herein discuss the mechanism of ETS and reflect on its prospects.

ETS for healthy volunteers

There have been several reports of taste-enhancing effects of ETS on healthy subjects. Nakamura et al. [9] used anodal ETS in six healthy university student volunteers to test the salt-taste-enhancing effect. The taste-enhancing effect of ETS was tested using 1-8% saline solution, with thinner concentrations having a stronger enhancing effect. The potentiation effect of ETS was also tested with 1-3 mA of electric direct current, and it was observed that the stronger the current, the stronger the potentiation effect. Sakurai et al. [14] used continuance square wave ETS in five healthy volunteers in their 20s to test the salt-taste-enhancing effect. All of them reported an increase in saltiness with ETS. Our report is unique in that it involved a real patient, not a healthy volunteer.

Mechanism of ETS

Various types of ETS have been described. Some use forward or backward [13] as well as alternating or direct current [15], and the waveform of current can vary [16]. We used a simple ETS in which 1 mA of direct current is passed through the skin to the chin and jaw (Figure 4). Studies are being conducted to determine what kind of ETS can modify and make tastes, but the mechanism is still largely unknown. Previous studies have stated that two possible mechanisms exist by which electrical stimulation from the skin increases or decreases taste [9].

The first hypothesis is that changes in taste occur through direct stimulation of taste-afferent nerve fibers. The electrical stimulation may directly affect nerve excitation [16]. In other words, the electrical current stimulates the sensory nerves and induces virtual taste, which increases the salty taste of the salt water, giving the illusion of a more pungent salty taste.

The second proposed mechanism is that the electric field generated by electrical stimulation creates a concentration gradient by electrophoresing taste substances such as sodium ions, creating a pseudo-tasty sensation. In the taste conduction pathway, the action of taste substances on channels or receptors on taste cells in the tongue's taste buds can be the trigger for gustatory nerve excitation. Electrical stimulation from the skin creates an electric field, which creates a concentration gradient of taste. As a result, certain areas of the tongue have a higher concentration of taste substances than food and drink that enter the oral cavity, and we may experience a falsely strong taste [17]. The concept of electric taste has also been proposed as having a unique flavor [9] and a virtual taste sense that can detect the taste of salt even in the absence of salt [18]. Therefore, ETS may act on a process different from conventional taste perception. Further research is required to gain more insights into this.

Comparison with tableware-like ETS device

Using our device, we applied the current directly to the jaw. On the other hand, ETS devices shaped like tableware have also been studied. The tableware-type device can be used naturally, without electrodes affixed to the body, and its utility has been reported [18].

ETS cannot deliver an electric current and enhance taste unless the electrodes and the food in contact with the electrodes are in contact with the body. Therefore, when using the ETS device shaped like tableware, we may only experience a taste change when we put the food in our mouth. In addition, since electrical stimulation is applied directly to the tongue and oral mucosa, we may be at risk of mucosal damage and may experience a bitter taste. The advantage of the proposed method of transcutaneous electrical stimulation to the chin and jaw over tableware-like ETS devices is the long duration of the effect and low possibility of mucosal damage. On the other hand, the disadvantage of our ETS with electrodes fixed to the skin is that fixing the electrodes requires time and effort. Further research is needed to determine what type of device is best suited.

Limitations of the report and recommendations for further research

The study is limited by its single-case design, which may restrict the generalizability of the findings. Including a larger sample size would enhance the robustness and validity of the results. We should confirm the generalizability not only for SAH patients but also for other stroke or other disease patients. The lack of a control group is another limitation, which precluded the ability to compare the effects of ETS on taste perception directly.

Besides, the study assessed taste perception at a single time point, thereby limiting insights into the long-term effects on salt intake habits, blood pressure trends, and hypertension-related events such as cerebrovascular disease. The purpose of this device is to allow patients with SAH to reduce salt consumption without affecting their enjoyment of food, ultimately enabling patients to lower their blood pressure. Since this study has only shown a strong perception of saltiness, future studies must study the actual salt intake and blood pressure as described above.

The presence or absence of side effects from ETS should also be considered. Finally, objective evaluations such as taste tests, including electrogustometry and taste disk method, as well as smell tests like the Alinamin test should be performed. This is because taste and smell disorders may be related to SAH [14,15] and should be objectively evaluated.

## Conclusions

This report demonstrates the efficacy of ETS in enhancing salt taste perception in SAH patients, and it is the first report of its kind involving an actual patient. Through psychophysical experiments, we showed that ETS effectively enhanced salt taste perception without altering salt concentration, suggesting its potential to reduce daily salt intake by enhancing salt taste perception. Further and more extensive studies are needed to validate these findings and explore the broader applicability of ETS in improving taste perception and adherence to dietary interventions while tracking blood pressure trends.
